# A Case of Cutaneous Leishmaniasis: Awareness and Recognition in Low-Endemic Areas

**DOI:** 10.7759/cureus.101349

**Published:** 2026-01-12

**Authors:** Garrett A Perchetti, Katherine Becker, Bianca Bicalho, Anubhav Poudel, Roxana Lazarescu

**Affiliations:** 1 Internal Medicine, Wyckoff Heights Medical Center, Brooklyn, USA; 2 Medicine, Touro College of Osteopathic Medicine, New York, USA

**Keywords:** cutaneous leishmaniasis, leishmania amastigotes, leishmania parasite infection, liposomal amphotericin b, neglected tropical diseases (ntds), ulcerative lesion

## Abstract

*Leishmania* is a parasitic organism that causes multiple clinical manifestations, including cutaneous leishmaniasis (CL), characterized by skin ulcers and satellite lesions. It is endemic to 98 countries, notably rural tropical regions. Although cutaneous forms are endemic to the United States, most cases occur among travelers who have traveled abroad. While international cases are well established, the United States demonstrates a substantial deficit in recognition and reporting of CL. We present the case of a 50-year-old woman who developed ulcerative lesions on the right auricle following an insect bite in Colombia. The patient was evaluated by multiple specialties over the course of one year before being diagnosed with CL. This case demonstrates a pattern of atypical presentations of leishmaniasis, with negative special stains on multiple specimens but detection by polymerase chain reaction (PCR). Our goal is to raise awareness of leishmaniasis in the United States and increase the use of PCR testing for diagnosis to make diagnosis and treatment more efficient.

## Introduction

Neglected tropical diseases are categorized by the World Health Organization as vector-borne diseases with reservoirs that maintain intricate life cycles, making it difficult to control their spread throughout the population [1]. Leishmaniasis, a group of diseases transmitted by the female sandflies Phlebotomus and Lutzomyia, is one of these neglected tropical diseases [2]. Leishmaniasis is endemic to 98 countries, with an estimated two million new cases each year [2]. Although most cases of leishmaniasis in the United States are acquired while traveling abroad, cutaneous forms endemic to Texas and Oklahoma exist [3]. The first reported case of autochthonous cutaneous leishmaniasis (CL) was in 1903, with an additional 80 cases diagnosed by 2020 [3]. Despite the well-known endemic prevalence of infection abroad, Texas is the only state required to report diagnoses [3], and only 20% of cases in the United States were reported between 2007 and 2023 [4]. This lack of reporting highlights the need for increased awareness of leishmaniasis diagnosis and treatment in the United States.

The parasitic organism Leishmania can cause three clinical prodromes, including CL, involving the skin ulcers, satellite lesions, and nodular lymphangitis; mucocutaneous leishmaniasis (MCL) with additional involvement of mucous membranes and connective tissue; and lastly visceral leishmaniasis (VL), which spreads to organs like the liver and spleen [2]. The parasite spreads to humans from the bite of a female sandfly, after which the protozoa enter phagolysosomes. CL occurs when the parasite enters and fills resident skin macrophages with amastigotes. These cells eventually burst and infect neighboring cells. VL occurs when amastigotes spread hematogenously to mononuclear cells in the liver, spleen, bone marrow, and intestinal lymph nodes [5].

Following a parasite inoculation, papules at the bite site are the first clinical sign of leishmaniasis. The growing papules combine to form well-defined ulcers, characterized by raised violaceous margins and epidermal disintegration [5]. These types tend to have a predilection for the face, ears, and extensor surfaces of the limbs. Histological examination, using formalin-fixed, paraffin-embedded tissue and Giemsa stain, is the current gold standard for diagnosis due to its high specificity. The classic organism can be seen as two to four mcm round or oval organisms with distinctive nuclei and kinetoplasts on skin scraping or biopsy. [2]. Differing methods suggest that samples taken from the ulcer borders yield the best results [5], whereas others state that the ulcer base is the optimal sampling site [2]. However, fine-needle aspiration is typically used because it provides improved patient comfort.

Herein, we discuss the case of a 50-year-old female presenting with chronic ulcerative lesions of the right auricle after sustaining an insect bite while in Colombia. The patient followed with multiple specialists in the United States for over one year before receiving the diagnosis of CL. While leishmaniasis is not widely endemic in the United States, there is a need for greater education and awareness among healthcare providers regarding the clinical signs of leishmaniasis to mitigate its spread and more efficiently provide treatment. Patient population demographics need to be considered when evaluating a presentation similar to leishmaniasis. Areas with high immigrant populations from endemic areas, such as Latin America or the Middle East, and previously employed military service members must have higher clinical suspicion for such a disease [3]. Heightened awareness is required regarding the potential rise in leishmaniasis incidence in the previously low-prevalence United States.

## Case presentation

**Figure 1:**
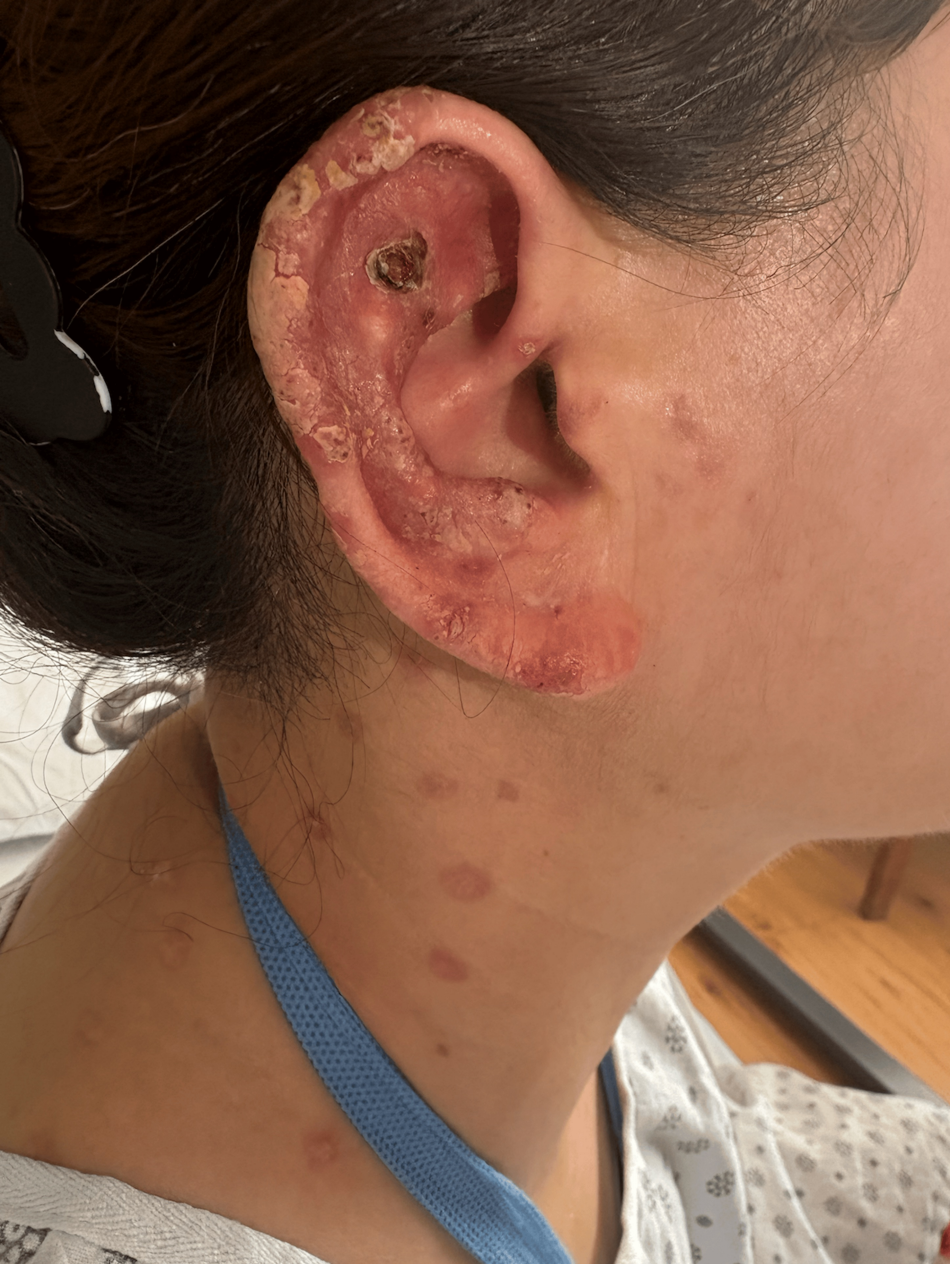
Right auricle with black necrotic ulcer and peripheral crusting lesions

A 50-year-old woman with a history of chronic hepatitis B virus infection, on long-term tenofovir therapy, presented to the emergency department after referral by her primary care physician for evaluation of a chronic ulcerative lesion of the right ear. On admission, her temperature was 36.4°C, pulse rate 76 bpm, respiratory rate 19 breaths per minute, blood pressure 115/88 mmHg, and oxygen saturation 96% on room air. On physical exam, the right auricle demonstrated a necrotic ulcer with thick yellow crusting and surrounding induration. Multiple small macules were observed to extend into the external auditory canal and on the upper back and neck (Figure 1). The oral exam was remarkable for a single black ulcer on the right buccal mucosa. A right submandibular lymph node was also appreciated.

Approximately one year before this presentation, while residing in Colombia, she recalled being bitten by an insect on the right auricle. Over the following months, it developed into a progressively worsening wound. The lesion became ulcerated and crusted, gradually increasing in size despite multiple courses of oral and topical medications prescribed by dermatologists (Figure 1).

Eight months prior to this hospitalization, the patient underwent multiple examinations by outpatient primary care and dermatology in attempts to control the lesions. An infectious disease panel, which included testing for *Treponema pallidum*, HIV, herpes simplex virus, varicella zoster virus, and tuberculosis, was negative. Bacterial and fungal cultures grew only Staphylococcus epidermidis, which was regarded as a contaminant. An initial shave biopsy of the skin demonstrated nonspecific acute and chronic inflammation with dermal fibrosis. During this time, the patient was treated with multiple topical steroids, topical and oral antibiotics, and intralesional Kenalog (ILK) injections. However, these did not result in clinically significant changes in the lesions. Further workup was ordered for evaluation since numerous attempts at clearing the ulcer with antibiotics and steroids proved ineffective.

The cutaneous shave biopsy was repeated three months later, which then revealed granulomatous inflammation and a lymphocytic infiltrate of CD3+ T cells and CD20+ B cells with immunohistochemical staining positive for polytypic kappa and lambda light chain expression. Gene rearrangement studies were further performed to determine lymphocyte clonality and to exclude a lymphoproliferative disorder. These test results together were consistent with a reactive process.

A rheumatologic workup was completed to rule out an autoimmune etiology, showing a low-titer positive ANA at 1:80, mildly positive anti-dsDNA antibody, and positive atypical p-ANCA. Testing for anti-MPO, anti-PR3, anti-SSA, anti-SSB, and anti-centromere antibodies was negative. Patch testing demonstrated nickel sensitivity, which was not believed to be clinically relevant.

Given the chronic ulcerative lesion of the right auricle and the history of an insect bite in an endemic area of *Leishmania*, CL was strongly suspected. The patient was admitted to the hospital and was started on empiric liposomal amphotericin B for seven days. She was premedicated with diphenhydramine and 0.9% normal saline, with renal function and electrolytes monitored daily.

While undergoing empiric treatment, a third biopsy of the right auricle was obtained for definitive diagnosis. The biopsy demonstrated necrotizing granulomatous inflammation with polymerase chain reaction (PCR) detection of *Leishmania*.

The patient tolerated the seven-day course of amphotericin well, with no evidence of acute kidney injury or electrolyte abnormalities. The right auricle showed improvement, with decreased inflammation, dried primary lesions, and no new lesions. She was discharged with instructions to follow up with ENT in one week.

## Discussion

This case highlights several important issues in the diagnosis and management of complex ulcerative cutaneous lesions in a non-endemic context, with eventual molecular confirmation of CL and empiric systemic treatment with liposomal Amphotericin B. It offers insights into diagnostic delays, the role of molecular testing, therapeutic choices, and challenges related to immunomodulation and comorbidities.

Diagnostic considerations and delay to diagnosis

Our patient had a protracted course of a chronic ulcer of the ear and auricular region, initiated after a presumed insect bite in Colombia. The differential diagnosis was broad: granulomatous inflammation on biopsy, negative bacterial/fungal/AFB stains, negative clonality studies, a low-titer ANA, p-ANCA positivity (with negative MPO/PR3), and patch-test nickel sensitivity. This complexity delayed an infectious etiology from being front of mind. In non‐endemic settings, CL is often overlooked, which contributes to diagnostic delay. Indeed, the changing epidemiology of leishmaniasis demonstrates that cases imported into non‐endemic regions may present atypically or with delayed recognition [6].

The utility of molecular diagnostics in this context is underscored: PCR detection of *Leishmania* is more sensitive than histology alone, and species or genotype identification via PCR or sequencing adds precision [7]. In this case, the biopsy from the auricle showed necrotizing granulomatous inflammation with multiple negative special stains, but only *Leishmania* was detected by *Leishmania* PCR (mini exon-1 DNA). This pattern is consistent with other reports of atypical or indolent presentations, in which conventional microscopy/stains may be negative or inconclusive. The anatomical location (ear/auricle, external canal) and mucosal involvement (oral ulcer on the buccal mucosa) raise concern for more than a simple cutaneous disease, suggestive perhaps of mucocutaneous involvement. The fact that the patient had an endemic exposure (Colombia) further supports the diagnosis. The presence of granulomatous inflammation with a reactive lymphocytic infiltrate (CD3+, CD20+, polytypic) also speaks against a primary lymphoproliferative process and supports a chronic inflammatory/infectious cause.

Therapeutic strategy and rationale

Once CL was suspected, the choice to initiate systemic liposomal amphotericin B was made empirically; one element was the patient’s complex presentation. The lesion involved the face/ear and extended into the external auditory canal; there was mucosal involvement (oral ulcer); prior topical and intralesional therapies had failed. Such features (facial lesion, mucosa involvement, chronicity) often prompt systemic therapy rather than just local therapy or topical/intralesional treatment.

Although most of the literature comes from endemic settings, L-AmB has been increasingly used in CL, particularly “complex” cases. A review noted cure rates of approximately 80-90% in mild-moderate CL with L-AmB [8]. The WHO/IDSA/ASTMH guidelines for leishmaniasis acknowledge L-AmB as one of several systemic options for CL/ML when systemic treatment is indicated [9]. Pharmacologically, liposomal amphotericin formulations reduce the toxicity of deoxycholate amphotericin, permitting safer systemic administration [10].

Given the long duration and failure of many prior interventions, treatment akin to that in endemic "complex" cases seemed reasonable. The patient was pretreated with a fluid bolus and antihistamine (diphenhydramine), and renal function and electrolytes were monitored, as appropriate, given the known risks of amphotericin therapy. Indeed, in prior reports, adverse effects (hypokalemia, rises in pancreatic or liver enzymes) with L-AmB in CL have been described. However, the drug is overall safe compared to older agents [11].

A point of interest is that in our case, a seven-day course was used (150 mg IV daily for seven days). There is heterogeneity in dosing regimens for L-AmB in CL. Some sources cite three mg/kg/day for eight days, as in one case report, for CL [12]. No randomized trials define the optimal dose in CL; many rely on observational data [13]. The patient’s dosing was weight-based and within commonly reported ranges, and renal function was normal at baseline and monitored closely throughout therapy, with no evidence of nephrotoxicity. Accordingly, our regimen, though empirical, is within the realm of reasonable systemic therapy.

Unique aspects and complicating factors in this case

Lesions of the ear and face raise an increased risk of disfigurement and may require more aggressive therapy. Many CL series focus on extremities; head/neck lesions can be more challenging to evaluate. The patient has chronic hepatitis B (on tenofovir). While this likely did not directly predispose to leishmaniasis, it raises considerations: the immunologic milieu may be altered, and potential hepatic/renal comorbidities must be considered when using amphotericin. Tenofovir therapy for chronic hepatitis B is not immunosuppressive and is unlikely to have influenced the progression of CL.

In contrast, repeated local immunosuppression with topical corticosteroids and intralesional triamcinolone may have impaired local immune responses, altered lesion morphology, and reduced diagnostic yield on early biopsy. Additionally, chronic HBV infection and nonspecific autoimmune serologies may have biased clinicians toward inflammatory etiologies, contributing to diagnostic delay. At baseline, she appears to have tolerated therapy without significant renal/hepatic adverse effects.

The patient’s year of progressive ulceration despite multiple topical and systemic treatments underscores how CL can masquerade as chronic dermatitis/infection. Early misclassification (topical steroids, ILK injections, systemic steroids) likely delayed diagnosis and may have worsened the lesion due to immunosuppression. Importation to non-endemic regions: This case occurred in the United States (New York City) in a patient with prior travel/residence in Colombia. That underscores the need for heightened awareness of CL in non-endemic settings.

The literature notes increasing recognition of imported cases and even local cases in the United States and Europe [6]. The case reinforces the diagnostic value of PCR for *Leishmania* (mini-exon 1 DNA). Given that conventional histology and stains were negative, the positive PCR result on day six of empiric therapy provided confirmation and will aid eventual species identification if performed. In non-endemic settings, laboratories may lack experience, and turnaround may be long; empiric therapy may thus be justified.

Limitations and considerations

Species identification has implications for prognosis, treatment response, and risk of mucocutaneous progression. Some species (e.g., the New World L. braziliensis complex) are at higher risk of mucosal spread and may warrant more aggressive therapy. The absence of that information thus limits targeted management. The empiric initiation of therapy prior to PCR results is defensible. Still, the exact sensitivity/specificity of the PCR in this case is unknown, and the possibility of false positivity (or incidental detection) should be considered, albeit unlikely given the clinical context.

The outcome at discharge was that the lesion was “dried, with no new lesions,” but there was not full resolution; longer-term follow-up is critical given the risk of relapse or mucosal dissemination, particularly given the unusual site (ear) and potential mucosal involvement. There is no mention of species-specific susceptibility or pharmacogenomics; as noted, treatment responses vary by species [14].

Implications for practice and further research

Clinicians practicing in non-endemic regions should consider CL in patients with chronic nonhealing ulcers at unusual sites, especially those with travel/residence in endemic regions or a history of insect bites. A delay in recognition may permit substantial tissue damage. Incorporation of early travel and exposure history into the diagnostic algorithm for chronic nonhealing ulcers, prompt consideration of CL in patients with granulomatous histology and negative routine microbiologic studies, and early use of *Leishmania* PCR or referral to reference laboratories would be useful in non-endemic settings. We also highlight the importance of clinician education, particularly for dermatology, primary care, and infectious disease providers in non-endemic regions, regarding imported leishmaniasis and the risks of empiric immunosuppression before infectious causes are excluded.

This case supports initiating systemic therapy for facial/auricular lesions with mucosal involvement or chronicity. Given the limited evidence, further data on optimal regimens (dose, duration) of L-AmB in CL are required. Outcomes from real-world observational series suggest 80-90% cure rates, but randomized trials are lacking [8]. Species-specific considerations: Identification of species might guide the aggressiveness of therapy (e.g., risk of mucosal involvement). Research into species-specific treatment response is ongoing [14].

Although our patient was apparently immunocompetent, the prolonged prior immunosuppression (systemic steroids) may have contributed to lesion chronicity. Future reports should address the impact of prior immunosuppression, host comorbidities (HBV, etc.), and lesion location on outcomes. The logistical aspects of diagnosis (reference labs, PCR turnaround) and therapy (availability of L-AmB, monitoring in non-tropical settings) merit attention. This case illustrates that with appropriate referral and multidisciplinary coordination (dermatology, ID, ENT, pathology, and molecular diagnostics), successful management is feasible.

## Conclusions

This case of chronic ulceration of the right ear, ultimately diagnosed as CL over the course of one year, underscores the diagnostic complexity and therapeutic challenges of leishmaniasis in non-endemic settings, such as the United States. Leishmaniasis is likely underreported in the United States, potentially due to barriers to diagnosis and limited awareness of atypical anatomical presentations. Key lessons from this case include recognizing diverse locations of cutaneous manifestations, conducting early molecular diagnostic studies when these sites are identified, and the role of systemic liposomal amphotericin B in complex CL. While further research is needed to define optimal treatment regimens across diverse clinical contexts, this case demonstrates a unique presentation and will help draw attention to these clinical barriers.

## References

[1] (2025). Neglected tropical diseases. Published.

[2] de Vries HJ, Schallig HD (2022). Cutaneous leishmaniasis: A 2022 updated narrative review into diagnosis and management developments. Am J Clin Dermatol.

[3] Curtin JM, Aronson NE (2021). Leishmaniasis in the United States: emerging issues in a region of low endemicity. Microorganisms.

[4] Panlilio M, Martini O, Tchernogorova E, Carboni A, Duffle D, Torgerson L (2025). Rising leishmaniasis cases in the United States based on registry data from 2007 to 2023 and the vital role of health care providers in awareness and management. JMIR Dermatol.

[5] Maxfield L, Crane JS (2020). Leishmaniasis. StatPearls [Internet].

[6] Rocha R, Pereira A, Maia C (2022). Non-endemic Leishmaniases reported globally in humans between 2000 and 2021: a comprehensive review. Pathogens.

[7] Galluzzi L, Ceccarelli M, Diotallevi A, Menotta M, Magnani M (2018). Real-time PCR applications for diagnosis of leishmaniasis. Parasit Vectors.

[8] Chivinski J, Nathan K, Naeem F, Ekmekjian T, Libman MD, Barkati S (2023). Intravenous liposomal amphotericin B efficacy and safety for cutaneous and mucosal leishmaniasis: a systematic review and meta-analysis. Open Forum Infect Dis.

[9] Aronson N, Herwaldt BL, Libman M (2016). Diagnosis and treatment of leishmaniasis: clinical practice guidelines by the Infectious Diseases Society of America (IDSA) and the American Society of Tropical Medicine and Hygiene (ASTMH). Clin Infect Dis.

[10] Shirzadi MR (2019). Lipsosomal amphotericin B: a review of its properties, function, and use for treatment of cutaneous leishmaniasis. Res Rep Trop Med.

[11] Wortmann G, Zapor M, Ressner R (2010). Lipsosomal amphotericin B for treatment of cutaneous leishmaniasis. Am J Trop Med Hyg.

[12] Shah NN, Nanjappa S, Messina JI, Greene JN (2016). Cutaneous leishmaniasis successfully treated by liposomal amphotericin B: a case report and review of the literature. Infectious diseases in clinical practice.

[13] Ubals M, Bosch-Nicolau P, Sánchez-Montalvá A (2021). Treatment of complex cutaneous leishmaniasis with liposomal amphotericin B. Pathogens.

[14] Madusanka RK, Silva H, Karunaweera ND (2022). Treatment of cutaneous leishmaniasis and insights into species-specific responses: a narrative review. Infect Dis Ther.

